# Biodegradability and Cytocompatibility of 3D-Printed Mg-Ti Interpenetrating Phase Composites

**DOI:** 10.3389/fbioe.2022.891632

**Published:** 2022-06-28

**Authors:** Xixiang Yang, Wanyi Huang, Desong Zhan, Dechun Ren, Haibin Ji, Zengqian Liu, Qiang Wang, Ning Zhang, Zhefeng Zhang

**Affiliations:** ^1^ School and Hospital of Stomatology, China Medical University, Liaoning Provincial Key Laboratory of Oral Diseases, Shenyang, China; ^2^ Shi-Changxu Innovation Center for Advanced Materials, Institute of Metal Research, Chinese Academy of Sciences, Shenyang, China

**Keywords:** 3D printing, Mg-Ti composite, degradation, Mg^2+^, MC3T3-E1 cells

## Abstract

Orthopedic hybrid implants combining both titanium (Ti) and magnesium (Mg) have gained wide attraction nowadays. However, it still remains a huge challenge in the fabrication of Mg-Ti composites because of the different temperatures of Ti melting point and pure Mg volatilization point. In this study, we successfully fabricated a new Mg-Ti composite with bi-continuous interpenetrating phase architecture by infiltrating Mg melt into Ti scaffolds, which were prepared by 3D printing and subsequent acid treatment. We attempted to understand the 7-day degradation process of the Mg-Ti composite and examine the different Mg^2+^ concentration composite impacts on the MC3T3-E1 cells, including toxicity, morphology, apoptosis, and osteogenic activity. CCK-8 results indicated cytotoxicity and absence of the Mg-Ti composite during 7-day degradation. Moreover, the composite significantly improved the morphology, reduced the apoptosis rate, and enhanced the osteogenic activity of MC3T3-E1 cells. The favorable impacts might be attributed to the appropriate Mg^2+^ concentration of the extracts. The results on varying Mg^2+^ concentration tests indicated that Mg^2+^ showed no cell adverse effect under 10-mM concentration. The 8-mM group exhibited the best cell morphology, minimum apoptosis rate, and maximum osteogenic activity. This work may open a new perspective on the development and biomedical applications for Mg-Ti composites.

## 1 Introduction

In the last few decades, there has been an overwhelming increase in the research on medical devices and implants because of aging population and ever-increasing human life expectancy. In particular, in the field of orthopedics, the number of orthopedic implant surgeries is constantly increasing. Ideal orthopedic implant materials should be outlined with the following characteristics (Witte 2010; Attarilar et al., 2020a): 1) good biocompatibility, 2) sufficient mechanical strength without the stress shielding effect, and 3) biological activity to promote healing.

Metals, ceramics, polymers, and composites are the commonly used orthopedics. Among them, metals are most suitable for wide applications in clinical conditions (Kandala et al., 2021). Ti and its alloys represent a feasible choice among materials for metallic orthopedic implants because of their satisfactory biocompatibility, high corrosion resistance, and excellent mechanical properties (Geetha et al., 2009; He et al., 2015; Zhu et al., 2016). Despite the mentioned advantages, their further clinical application remains a challenge due to some major drawbacks such as the stress shielding effect and being biologically inert (Stanec et al., 2016; Bobbert et al., 2017). Various methods of fabricating less stiff modulus orthopedic implants have been reported (Ouyang et al., 2019; Esen et al., 2020; Liang et al., 2021; Xu et al., 2021), and the control of porosity is considered a promising method (Meenashisundaram et al., 2020; Zhang et al., 2020; Zhang et al., 2021). On one hand, the porous structure of Ti-based materials effectively reduced the stress shielding effect between the implant and the surrounding bone. On the other hand, it can induce blood supply to the scaffold to supply oxygen and nourishment needed for tissue repair, promoting the generation and calcification of new bone tissues (Liu et al., 2015; Claros et al., 2016; de Krijger et al., 2017). Furthermore, the bioactivity of Ti and Ti alloys can also be improved by adding other elements, such as magnesium and zinc, into the porous structure (Wong et al., 2017; Yao et al., 2022).

Recently, Mg and Mg alloys have been widely designed and reported as potential biodegradable orthopedic implant materials (Witte et al., 2005). Compared with other biomedical metals, Mg-based implants have become increasingly attractive because of their appropriate mechanical properties, such as low density, high specific strength, and low elastic modulus (Mps et al., 2006). The elastic modulus of Mg is close to that of the bone, and it can effectively decrease the stress shielding effect at the bone-implant interface (Li et al., 2016; Wang N et al., 2020). Moreover, as one of the most abundant elements in the human body, Mg can be degraded and absorbed along with the human body’s self-healing. In addition, the functional effects of Mg include regulating bone metabolism, stimulating new bone formation, and increasing bone cell adhesion (Li et al., 2018).

For the aforementioned reasons, the combination of Ti and Mg seems a promising new idea for fabricating ideal biomedical materials. Martin et al. and Balog et al., (2019) successfully produced Ti+ (12, 17, and 24 vol.%) Mg composites by powder metallurgy. The mechanical and bioactive properties of the composites demonstrated immense potential for application as dental implants. However, Mg filaments were arrayed along the extrusion direction and embedded in the Ti matrix. In the case of low Mg content, the filaments could barely connect with each other. Ouyang et al. (2019) reported that a new Mg-Ti composite was manufactured through the process of spark plasma sintering (SPS). Despite the composite exhibiting good mechanical properties, the Mg-rich regions were non-uniformly distributed among the Ti matrix. In general, it is difficult to achieve ideal Mg-Ti composite biomedical materials using the traditional casting methods. In this context, 3D printing offers many advantages in manufacturing orthopedic implants, including free designation and high precision (Campanelli et al., 2017). Three-dimensional printing takes full advantage of the possibilities to realize the addition of functional elements in Ti alloys and the processing of implants with porous structures (Attarilar et al., 2020b; Fan et al., 2021). The type of repeating unit cell and its dimensions can be chosen to adjust the mechanical properties of the porous biomaterials to achieve an excellent match for the mechanical properties of bone (Eltorai, Nguyen and Daniels 2015; Stanec et al., 2016).

In our previous study (Zhang et al., 2020), we proposed a new fabrication approach to create a good combination of properties in a Mg-NiTi composite. This approach is also applicable in engineering other material systems to improve performance. In the present study, a pure Ti scaffold with three-dimensional (3D) interpenetrating phase architecture was fabricated by the 3D printing technology. We fabricated a Mg-Ti composite by pressureless infiltration of the Mg melt into the scaffold. The degradation behavior of the Mg-Ti interpenetrating phase composite *in vitro* and the bio-compatibility of the Mg-Ti composite degradation process were evaluated. Then, the effect of Mg^2+^, produced in the degradation process, on MC3T3-E1 cells was investigated by various Mg^2+^ concentration tests.

## 2 Materials and Methods

### 2.1 Material Preparation and Characterization

The fabrication methods of the Mg-Ti composite can be seen in our previous study (Zhang et al., 2020). In the following tests, we chose commercial pure Mg as the control material. Scanning electron microscope (SEM, Zeiss Merlin Compact, Zeiss, Germany) imaging coupled with energy-dispersive spectroscopy (EDS) analysis was performed to characterize the microstructure of the Mg-Ti composite and pure Mg specimens. All the specimens with a size of φ10 mm × 2 mm were prepared for the *in vitro* studies and immersion tests. Silicon carbide (SiC) papers were used to polish the samples to 800–2000 grit. Then, the disc samples were cleaned with distilled water for 10 min and sterilized with ultraviolet for 40–60 min, following ultrasonic etching in acetone and ethyl alcohol.

### 2.2 Immersion Test

All the samples for the immersion test were placed in 12-well cell culture plates and immersed in modified Eagle’s medium alpha (α-MEM) supplemented with 10% fetal bovine serum (FBS) at 37°C for 1, 3, 5, and 7-days. The immersion ratio was fixed as 1.25 cm^2^/ml with the α-MEM medium refreshed every 24 h. The medium pH value at regular time points was determined by using a pH detection device (PHS-3C, Leica, China). The corrosion products produced in the process of degradation were dried by hot air, following the removal with a chromic acid solution (200 g/L CrO_3_+ 10 g/L AgNO_3_). Then, the samples’ morphology was assessed by using a digital camera, and the microstructure was analyzed by SEM and EDS.

### 2.3 *In Vitro* Cell Tests

#### 2.3.1 Extract Preparation and Cell Culture

The extracts were used for the *in vitro* tests. After 1, 3, 5, and 7-days of immersion, the Mg-Ti composite and pure Mg samples were immersed in an α-MEM medium containing 10% FBS for 72 h at 37°C in a humidified atmosphere of 5% CO_2_. The immersion ratio was selected, as mentioned previously according to the standard ISO 10993. After filtrating with a filter (0.22 μm), the extracts were collected and then diluted six times with the α-MEM medium for *in vitro* tests.

MC3T3-E1 cells were chosen to test cell morphology, proliferation, apoptosis, and differentiation. The cells were cultured in α-MEM supplemented with 10% FBS and 1% penicillin and streptomycin in a humidified atmosphere at 37°C with 5% CO_2_. When the monolayer reached sub-confluence, the cells were subcultured with 0.25% trypsin.

#### 2.3.2 Cell Proliferation and Cytotoxicity Test

Cell-Counting Kit-8 (CCK-8, United States Everbright Inc., Silicon Valley, United States) assay was chosen to evaluate the effects of the Mg-Ti composite and pure Mg extracts on cell proliferation. Cells were seeded in 96-well plates at 3 × 10^3^ cells/well for 24 h. They were washed twice with PBS, and the medium was replaced by 100 μl extracts or a normal culture medium after 24 h of attachment. After 1, 2, and 3-days of culturing, 100 μl α-MEM with 10% CCK-8 was added after rinsing twice with PBS; then, the plate was incubated for 2 h at 37°C. The 450 nm optical density was measured by using a microplate reader (Infinite M200, Tecan, Austria). Three replicates were chosen per group.

#### 2.3.3 Cell Morphology Staining

To detect the effect of the Mg-Ti composite and pure Mg extracts on cell morphology, MC3T3-E1 cells were incubated on a 24-well cell culture plate with the diluted extracts at a density of 1 × 10^4^ per well for 4 and 24 h. At each time point, the cells were permeabilized with 0.1% Triton X-100 after washing with PBS three times. Then, the permeabilized cells were supplemented with 100 nmol/L rhodamine-phalloidin (Cytoskeleton, Inc., Denver, CO, United States) for 30 min in the dark at room temperature. After that, the cells were stained with DAPI for 2 min coupled with washing with PBS three times before observation with a fluorescence microscope (ZEISS, Germany).

#### 2.3.4 Cell Apoptosis

An Annexin V-FITC/PI kit (United States Everbright Inc., Silicon Valley, United States) was used to detect the effects of the sample extracts on cell apoptosis quantified through the standard flow cytometry test, according to the manufacturer’s protocol. MC3T3-E1 cells were seeded in 12-well plates at 1 × 10^5^ cells/well in a 1 ml medium for 24 h. The medium was replaced by prepared extracts or a normal culture medium, respectively, for 1 and 3-days. At each time point, the cells were digested with 0.25% trypsin and collected for the stain after washing with PBS three times. The collected cells were re-suspended with 100 μl binding buffer and stained with Annexin V-FITC and propidium iodide (PI) for 15 min in the dark. Before the flow cytometry (BD, LSRFortessa, United States) test, 300 μl binding buffer was added to each sample and blended evenly.

#### 2.3.5 Alkaline Phosphatase (ALP) Activity

MC3T3-E1 cells were seeded in 12-well plates at 1 × 10^5^ cells/well for 24 h. The culture medium was replaced with the prepared extracts, and the culture medium contained an osteogenesis-inducing component. The extracts were refreshed every 2-days. The activity of ALP was evaluated with an Alkaline Phosphatase Assay Kit, according to the manufacturer’s instructions (Beyotime, China) after culture for 7 and 14-days. The protein content was measured following the protocol of the BCA protein assay kit (Beyotime, China). The ALP activity test of all samples was normalized using the protein concentration.

### 2.4 Cell Responses to Varying Mg^2+^ Concentrations

#### 2.4.1 Preparation of the Medium With Varying Mg^2+^ Concentrations

To simulate the effect of 7-day degradation of Mg^2+^ production on MC3T3-E1 cells, we prepared α-MEM with varying Mg^2+^ concentrations. First, inductively coupled plasma mass spectrometry (7800 ICP-MS, Agilent, United States) was performed to detect the Mg^2+^ concentration of pure Mg and Mg-Ti composite extracts. Then, the sterilized Mg chloride solution was applied to elevate the α-MEM Mg^2+^ concentration, according to the result of the Mg^2+^ concentration test.

#### 2.4.2 Effect of Varying Mg^2+^ Concentrations on MC3T3-E1 Cells

CCK-8 assay was performed to evaluate the effects of varying Mg^2+^ concentrations on MC3T3-E1 cells in cytotoxic and proliferation ability. The cell morphology was stained with phalloidin to observe the effect of varying Mg^2+^ concentrations on the intracellular F-actin cytoskeletal network for 4 and 24 h. The cell apoptosis rate of MC3T3-E1 cells incubated with varying Mg^2+^ concentrations was determined by Annexin V-FITC/PI double staining for the period of 1 and 3-days. To determine the effect of varying Mg^2+^ concentrations on the osteogenic differentiation ability, an ALP assay was carried out after incubation for 7-days and 14-days.

### 2.5 Statistical Analysis

All experiments were repeated by at least three times for statistical purposes. Statistical analysis was performed with SPSS 25.0 software. Differences between the groups were analyzed by one-way analysis of variance (ANOVA) followed by Tukey’s test.

## 3 Results

### 3.1 Morphology and Microstructure Characterization

**FIGURE 1:**
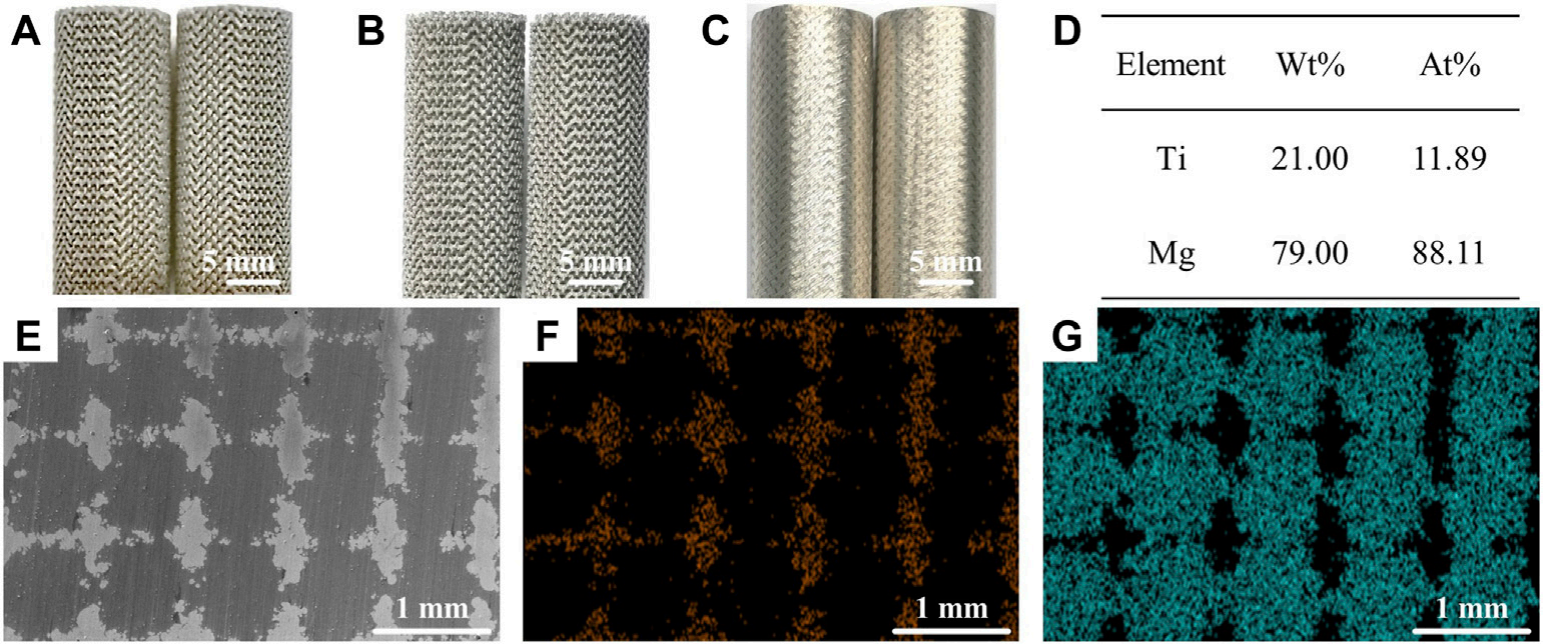
Morphology of the Mg-Ti composite. **(A–C) (A)** 3D-printed Ti scaffold. **(B)** Ti scaffold after acid treatment. **(C)** Mg melt infiltration into the Ti scaffold. EDS results **(D)** and the corresponding SEM images **(E–G)** of the Mg-Ti composite, **(F)** Ti, and **(G)** Mg.

Figure 1 shows the morphology and microstructures of the Mg-Ti composite. The microstructure and elemental composition of samples are analyzed *via* SEM coupled with EDS. SEM images show that the surface of the sample was smooth without obvious structural flaws, for example, pores or micro-cracks. It indicates that after the infiltration of the Mg melt into the Ti scaffold and subsequent solidification, the Mg-Ti composite materials are highly densified with the Mg and Ti phases interpenetrating the 3D space. In addition, the width of the Mg phase or the interspace between neighboring Ti struts is close to 700 μm in the sample. To further detect the element distribution, EDS is performed. The EDS analysis presents that Ti and Mg are the main elements in all tested specimens.

### 3.2 Immersion Test

**FIGURE 2:**
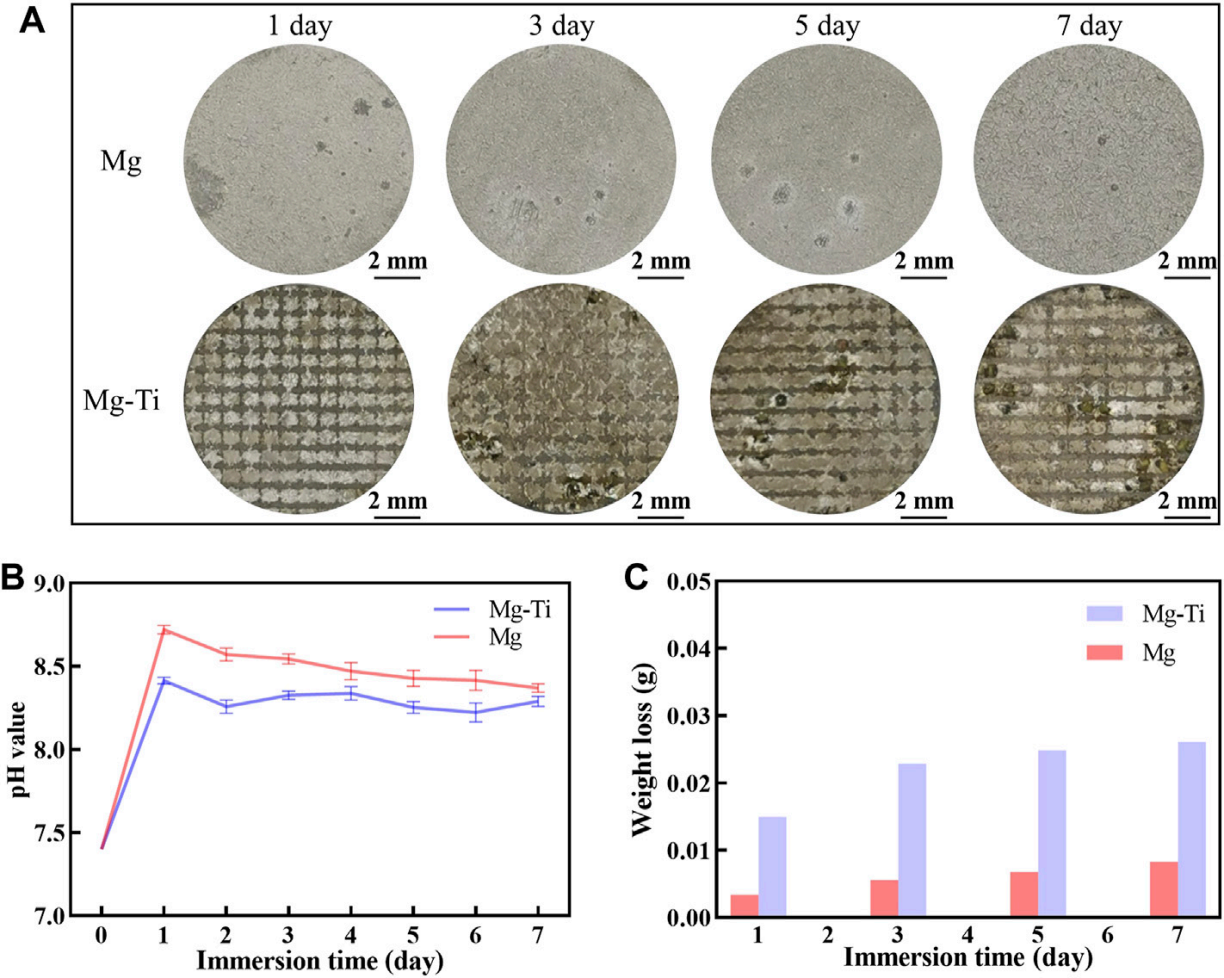
**(A)** Corroded surface photographs of pure Mg and Mg-Ti composite (corrosion products were removed). **(B)** pH values of pure Mg and the Mg-Ti composite in the α-MEM solution with 10% FBS at 37°C and 5% CO_2_. **(C)** Weight loss of pure Mg and Mg-Ti composite.

Figure 2 shows the results of pH value, weight loss, and macroscopic morphology of pure Mg and Mg-Ti composites. It is observed that the pH values of the Mg-Ti composite and pure Mg extracts both reached the maximum on the first day. The pH values of the Mg-Ti group are higher than those of the Mg group at all the test time points. The degradation of Mg proceeds gradually, slows down with the immersion time, and then becomes saturated after 7-days for both the Mg-Ti composite and pure Mg. The weight loss of the Mg-Ti composite is much higher than that of pure Mg after immersion for 7-days. It reaches a saturation point for the Mg-Ti composite and pure Mg after 5-days of degradation. The macroscopic appearance of the pure Mg and Mg-Ti composite shows that the degradation starts from the edge and extends toward the center of the materials with the increase in immersion time.

**FIGURE 3:**
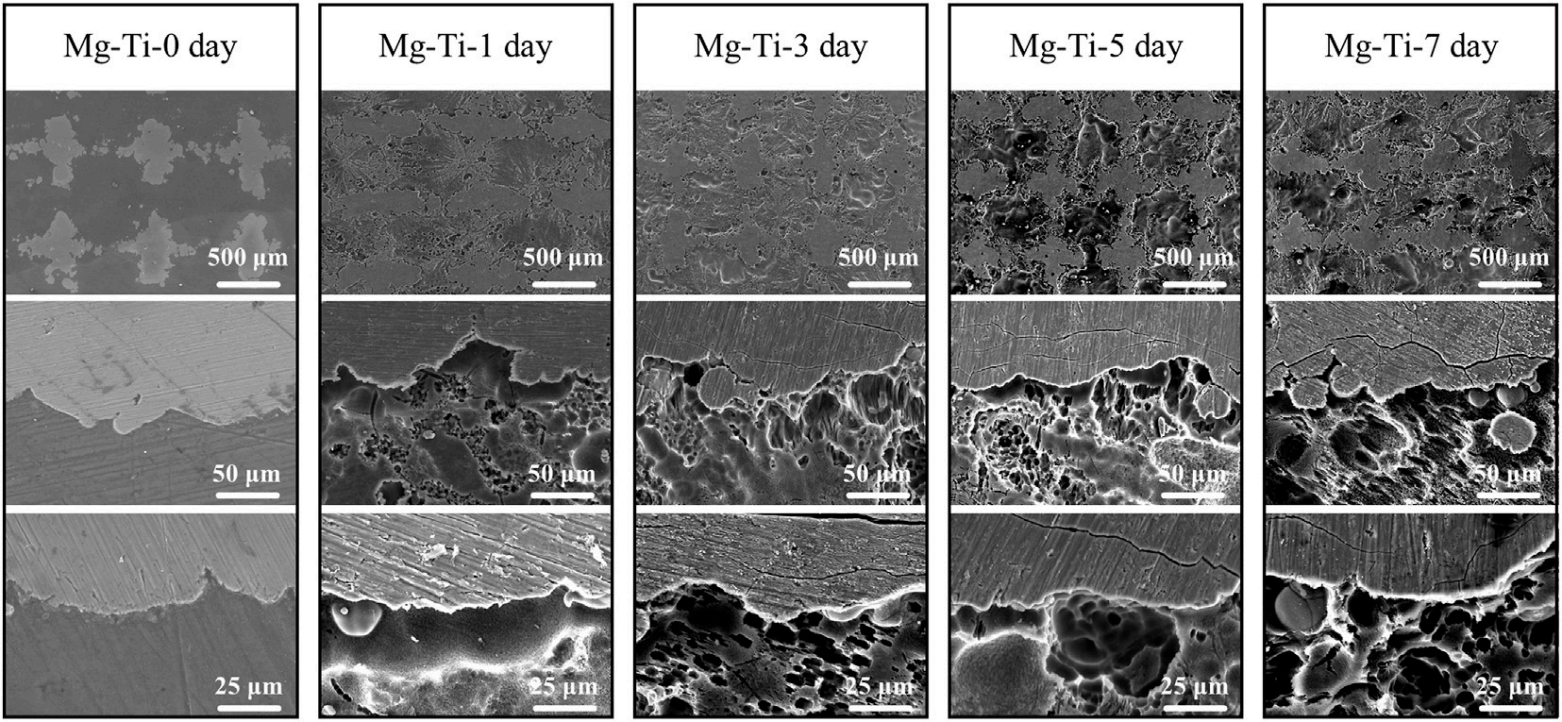
Microscopic morphology of the Mg-Ti composite after 0, 1, 3, 5, and 7 days of degradation (corrosion products were removed).

**FIGURE 4:**
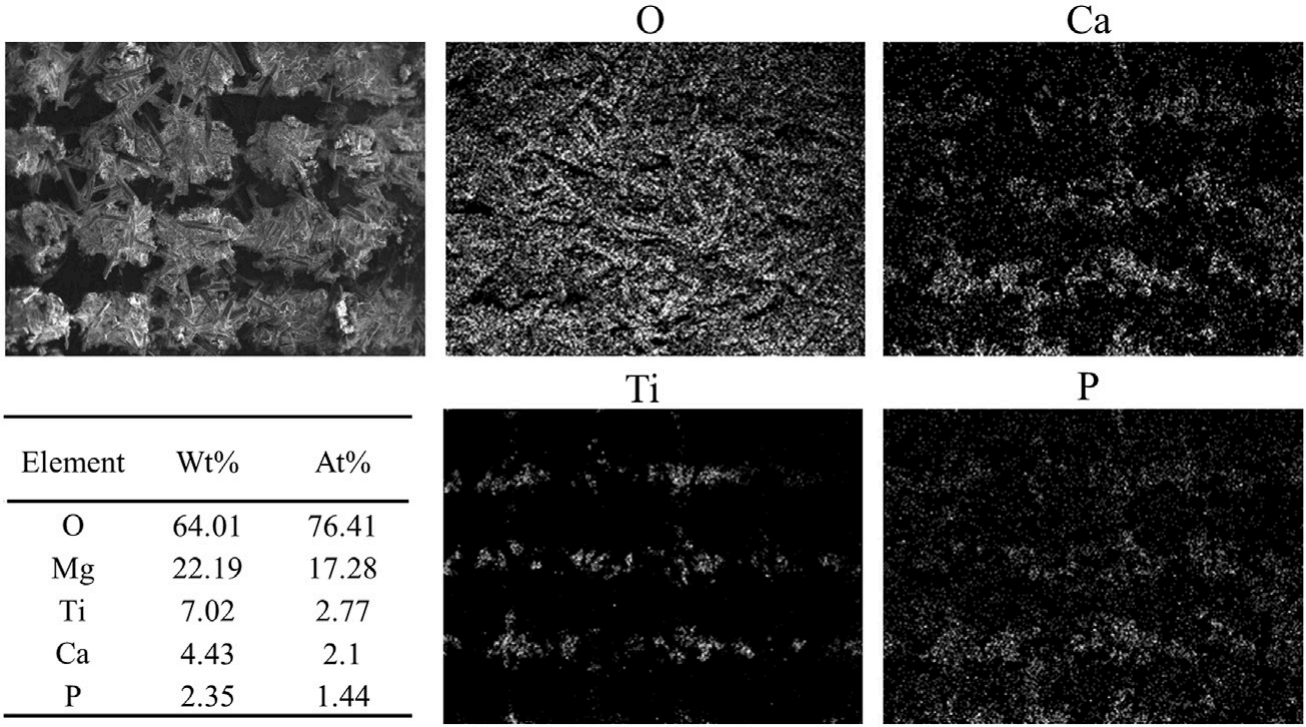
SEM images and EDS analysis of the Mg-Ti composite after 7 days of degradation.

Figure 3 shows the microscopic morphology of the Mg-Ti composite within 7-days of degradation. After removing the corrosion products, it can be observed that the Ti matrix in the Mg-Ti composite is almost integrated, whereas the Mg area is partially degraded. Several corrosion pits or cracks are basically observed in the Mg-rich area, whereas very few are observed in the Ti area. From the microscopic point of view, the degradation of the Mg-Ti composite starts from the interface of the Ti and Mg region, leading to a porous morphology of the corroded Mg area. With the prolongation of immersion time, the corroded area of the Mg region is extended and spreads to the central regions of Mg. Figure 4 shows the SEM and EDS images of the corrosion product of the Mg-Ti composite after 7-days of degradation. The Ca/P rate is close to 1.4.

### 3.3 *In Vitro* Cytocompatibility of MC3T3-E1 Cells

#### 3.3.1 Mg^2+^ Concentration of Extracts

**FIGURE 5:**
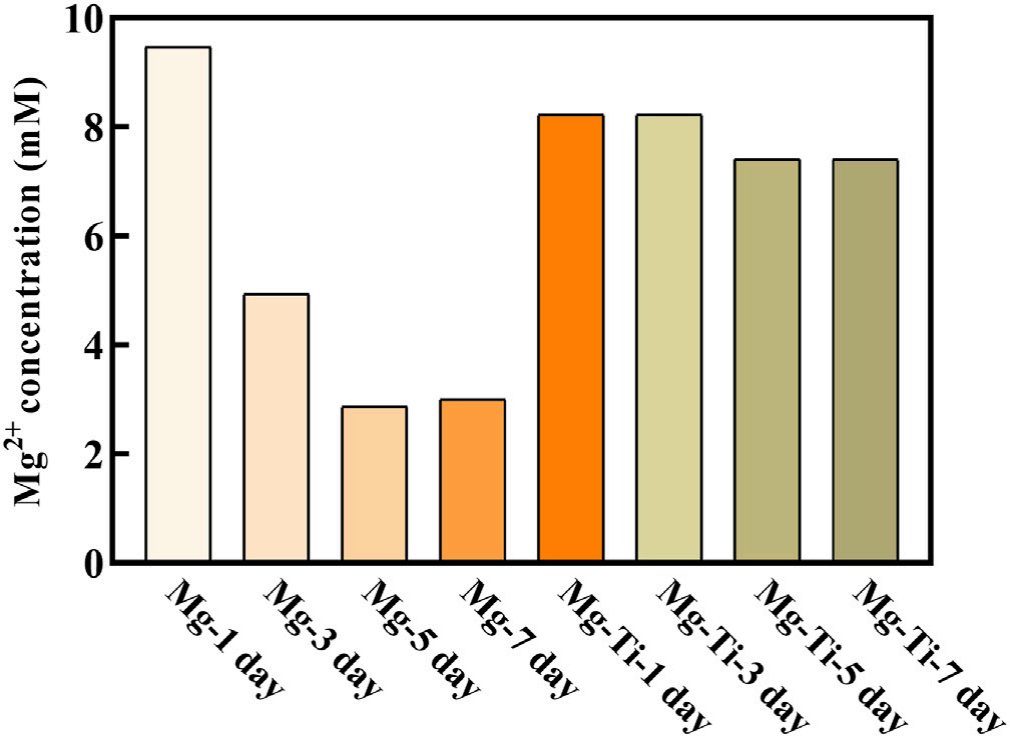
Extract of the Mg^2+^ concentration of the degraded Mg-Ti composite and pure Mg after diluting six times.

Figure 5 shows the Mg^2+^ concentration of pure Mg and the Mg-Ti composite extracts at different periods of immersion time. The Mg-1 day group exhibits the highest Mg^2+^ concentration (9.6 mM). With the immersion time prolonged, the Mg^2+^ concentration for the pure Mg groups decreases gradually and eventually stabilizes for 5-days (3 mM). For the Mg-Ti group, the Mg^2+^ concentration remains stable around 8 mM in the process of 7-days of degradation. Thereby, we selected a range of concentrations (2, 4, 6, 8, and 10 mM) to detect the effect of various Mg^2+^ concentrations on MC3T3-E1 cells.

#### 3.3.2 Cell Proliferation and Cytotoxicity

**FIGURE 6:**
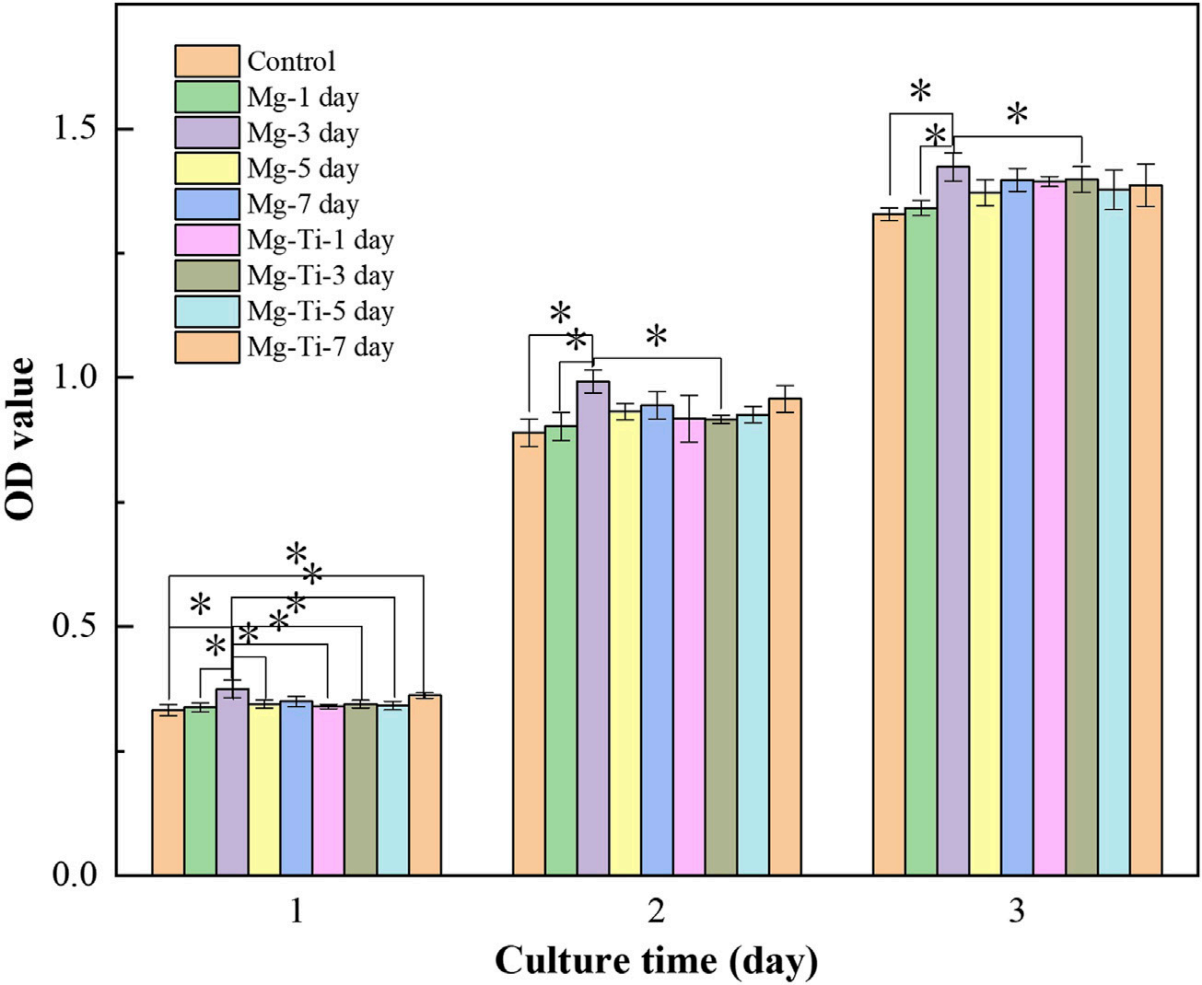
Optical density value (OD value) of MC3T3-E1 cells cultured with extracts of the degraded Mg-Ti composite and pure Mg.

**FIGURE 7:**
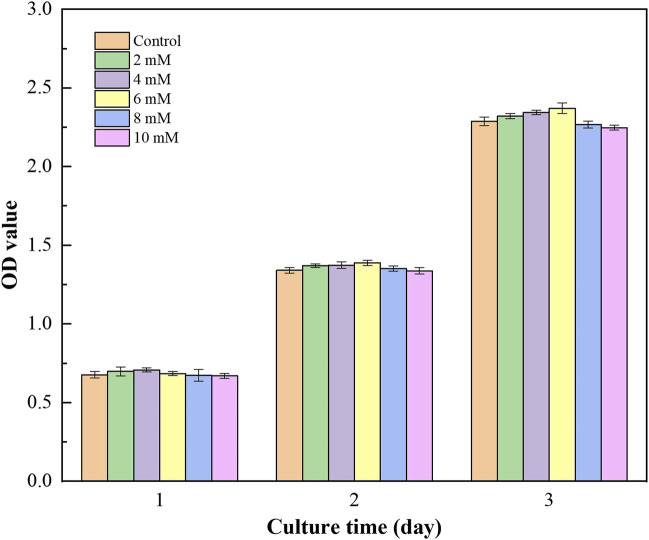
OD value of MC3T3-E1 cells cultured with gradient Mg^2+^ concentrations.

The CCK-8 results (Figure 6) show that the cells proliferate well in the pure Mg and Mg-Ti groups during incubation for 3-days. It displays that the OD values of all the groups increase gradually over the incubation time. Within 3-days of culture, the Mg-3 day group displays higher proliferation than the control group (*p* < 0.05). The relative growth rates (RGR) of MC3T3-E1 cells are shown in Table. 1. The RGR is calculated by a formula, according to the standard United States Pharmacopeia. The cell viability results of all specimens are more than 75%, indicating no cytotoxicity of all groups through 3 days of culture. As shown in Figure 7, the effects of Mg^2+^ concentrations on the cell proliferation ability are observed. It can be seen that the OD values gradually increased over the incubation time. From 1-day to 3-days of culture, all groups showed no statistical difference within the RGR ranging from 0–1 (no toxicity).

**TABLE 1:** Relative growth rate (RGR) and cytotoxicity level of MC3T3-E1 cells cultured with extracts of the degraded Mg-Ti composite and pure Mg at different detection periods.

Sample	1 day		2 days		3 days	
	RGR (%)	Grade	RGR (%)	Grade	RGR (%)	Grade
**Mg-1 day**	**101.88±0.01**	**0**	**101.42±0.03**	**0**	**100.89±0.01**	**0**
**Mg-3 days**	**112.89±0.02**	**0**	**111.56±0.02**	**0**	**107.12±0.03**	**0**
**Mg-5 day**	**103.80±0.01**	**0**	**104.77±0.02**	**0**	**103.21±0.03**	**0**
**Mg-7 day**	**105.40±0.01**	**0**	**106.15±0.03**	**0**	**105.18±0.02**	**0**
**Mg-Ti-1 day**	**102.40±0.01**	**0**	**103.23±0.05**	**0**	**104.92±0.01**	**0**
**Mg-Ti-3 day**	**103.77±0.01**	**0**	**102.97±0.01**	**0**	**105.22±0.03**	**0**
**Mg-Ti-5 day**	**102.95±0.01**	**0**	**104.08±0.02**	**0**	**103.66±0.04**	**0**
**Mg-Ti-7 day**	**108.97±0.01**	**0**	**107.60±0.03**	**0**	**104.33±0.04**	**0**

RGR, relative growth rate; Grade, the cytotoxicity level of MC3T3-E1 cells.

#### 3.3.3 Cell Morphology Staining

**FIGURE 8:**
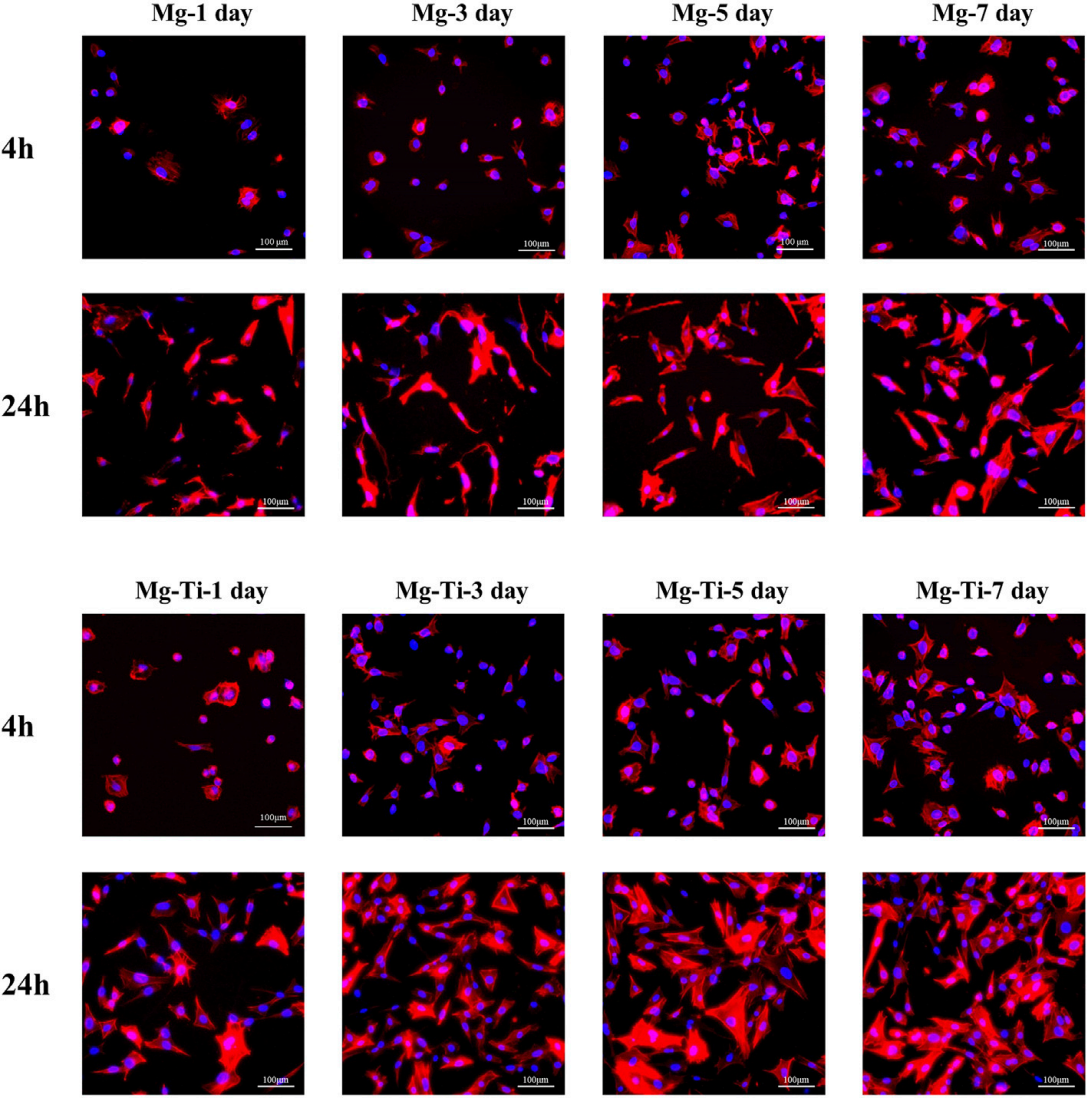
Morphology staining of MC3T3-E1 cells cultured with extracts of the degraded Mg-Ti composite and pure Mg for 4 and 24 h.

**FIGURE 9:**
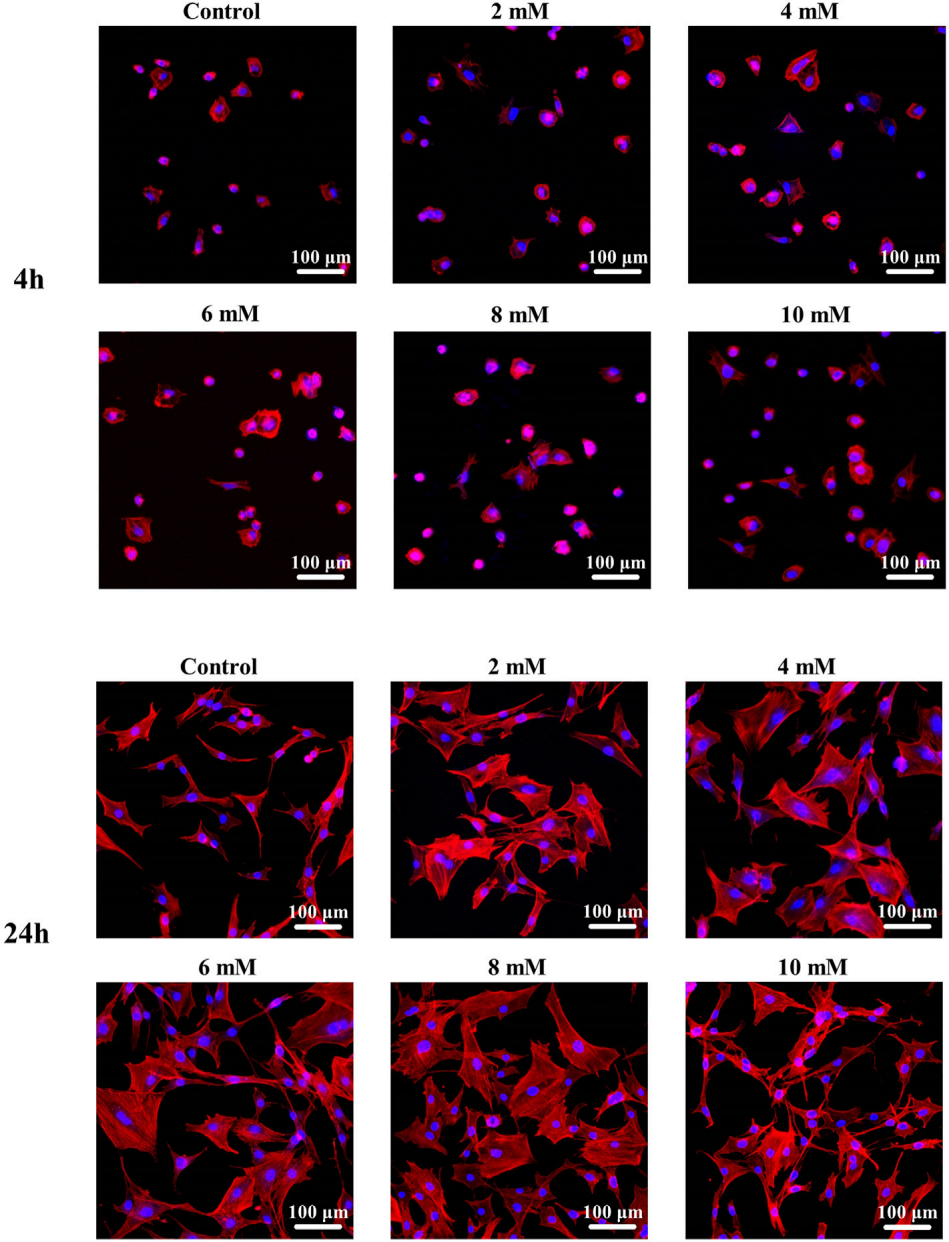
Morphological staining of MC3T3-E1 cells cultured with gradient Mg^2+^ concentrations for 4 and 24 h.

The fluorescence staining results of the intracellular F-actin cytoskeletal network with rhodamine-phalloidin and DAPI are shown in Figure 8. At the period of 4 h, the cells show obvious spindle morphology with little filopodia in both pure Mg and Mg-Ti groups. With the incubation time prolonged to 24 h, the cells present a fibrous structure with apparent F-actin, filopodia, and lamellipodia observed in the Mg-Ti group. Compared with pure Mg, cells provide more area in the Mg-Ti group, which suggests that the initial attachment behavior of MC3T3-E1 cells is better. As shown in Figure 9, the addition of Mg^2+^ promotes the expansion of MC3T3-E1 cells. Moreover, the cell exhibits a better shape configuration and displays better adhesion in the 8-mM group.

#### 3.3.4 Apoptosis Analysis

**FIGURE 10:**
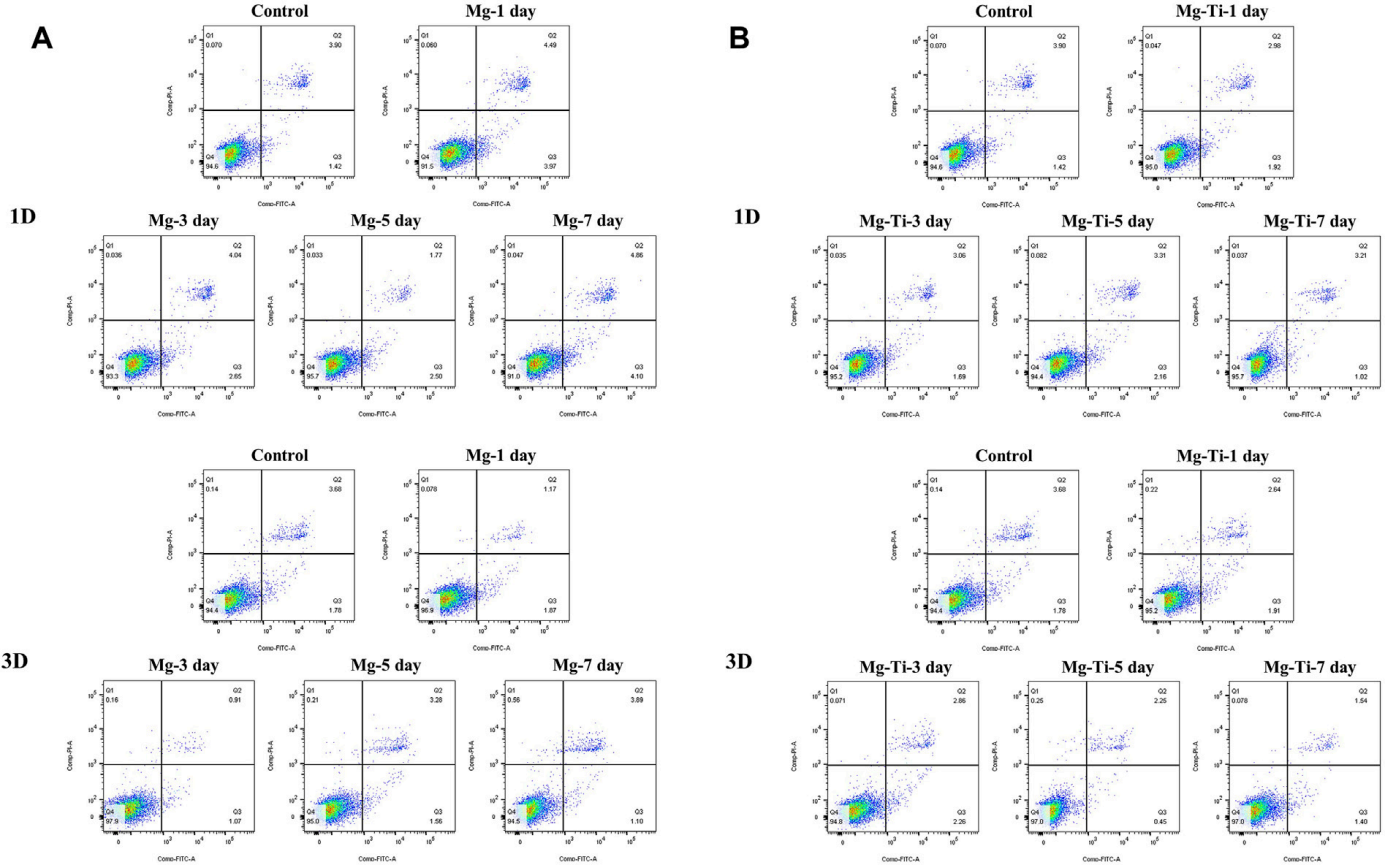
Apoptosis of MC3T3-E1 cells cultured with extracts of degraded pure **(A)** Mg and **(B)** Mg-Ti composite for 1 day and 3 days.

**FIGURE 11:**
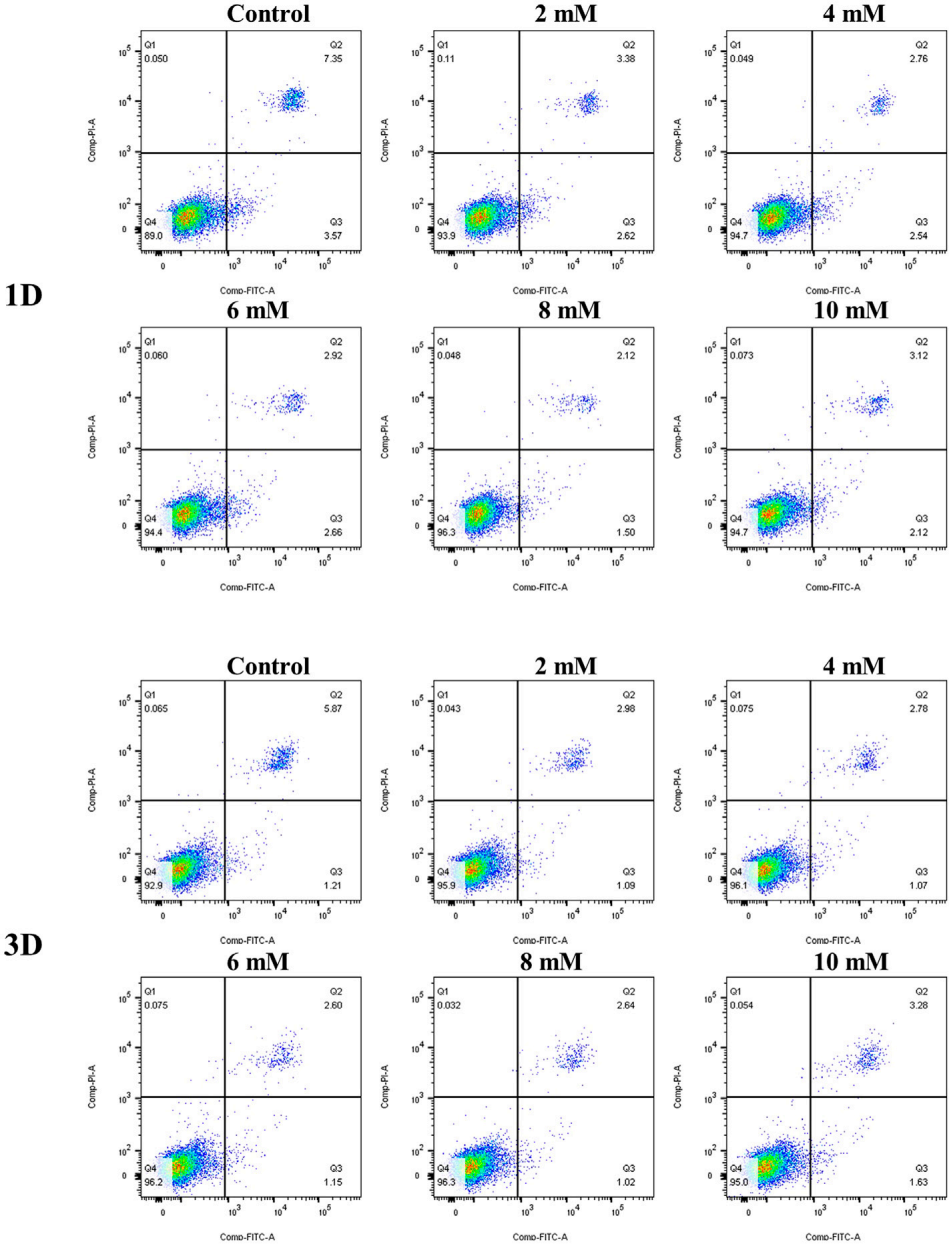
Apoptosis of MC3T3-E1 cells cultured with gradient Mg^2+^ concentrations for 1 day and 3 days.

The results of the cell apoptosis analysis are shown in Figure 10 and Figure 11. Apoptosis is an active biological mechanism leading to programmed cell death. The decrease in the apoptosis rate indicates an increase in the proliferative activity of MC3T3-E1 cells. Figure 10 demonstrates the cell apoptosis culture in pure Mg and Mg-Ti composite extracts with different degradation times. After seeding for 1-day and 3-days, the apoptosis of all groups including pure Mg and the Mg-Ti composite is less than 10%, which is an acceptable range. On day 1, the apoptosis of the Mg-Ti group is lower than that of the pure Mg group but is slightly higher than that of the control group. On day 3, the apoptosis rates of pure Mg and Mg-Ti groups are both lower than those of the control group (Table 2). The apoptosis results of MC3T3-E1 cells on varying Mg^2+^ concentrations within 3-days are shown in Figure 11. After 3-days of incubation, the addition of Mg^2+^ reduces the apoptosis of MC3T3-E1 cells with the 8 mM Mg^2+^ exhibiting minimum apoptosis.

**TABLE 2:** RGR and the cytotoxicity level of MC3T3-E1 cells cultured with gradient Mg^2+^ concentrations at different detection periods.

Sample (mM)	1 day		2 days		3 days	
	RGR (%)	Grade	RGR (%)	Grade	RGR (%)	Grade
**2**	**103.14±0.03**	**0**	**102.21±0.01**	**0**	**101.45±0.02**	**0**
**4**	**104.47±0.01**	**0**	**102.46±0.02**	**0**	**102.48±0.01**	**0**
**6**	**101.13±0.01**	**0**	**103.53±0.02**	**0**	**103.66±0.03**	**0**
**8**	**99.49±0.04**	**1**	**100.90±0.02**	**0**	**99.10±0.02**	**1**
**10**	**98.91±0.02**	**1**	**99.75±0.02**	**1**	**98.26±0.02**	**1**

RGR, relative growth rate; Grade, the cytotoxicity level of MC3T3-E1 cells.

#### 3.3.5 ALP Activity

**FIGURE 12:**
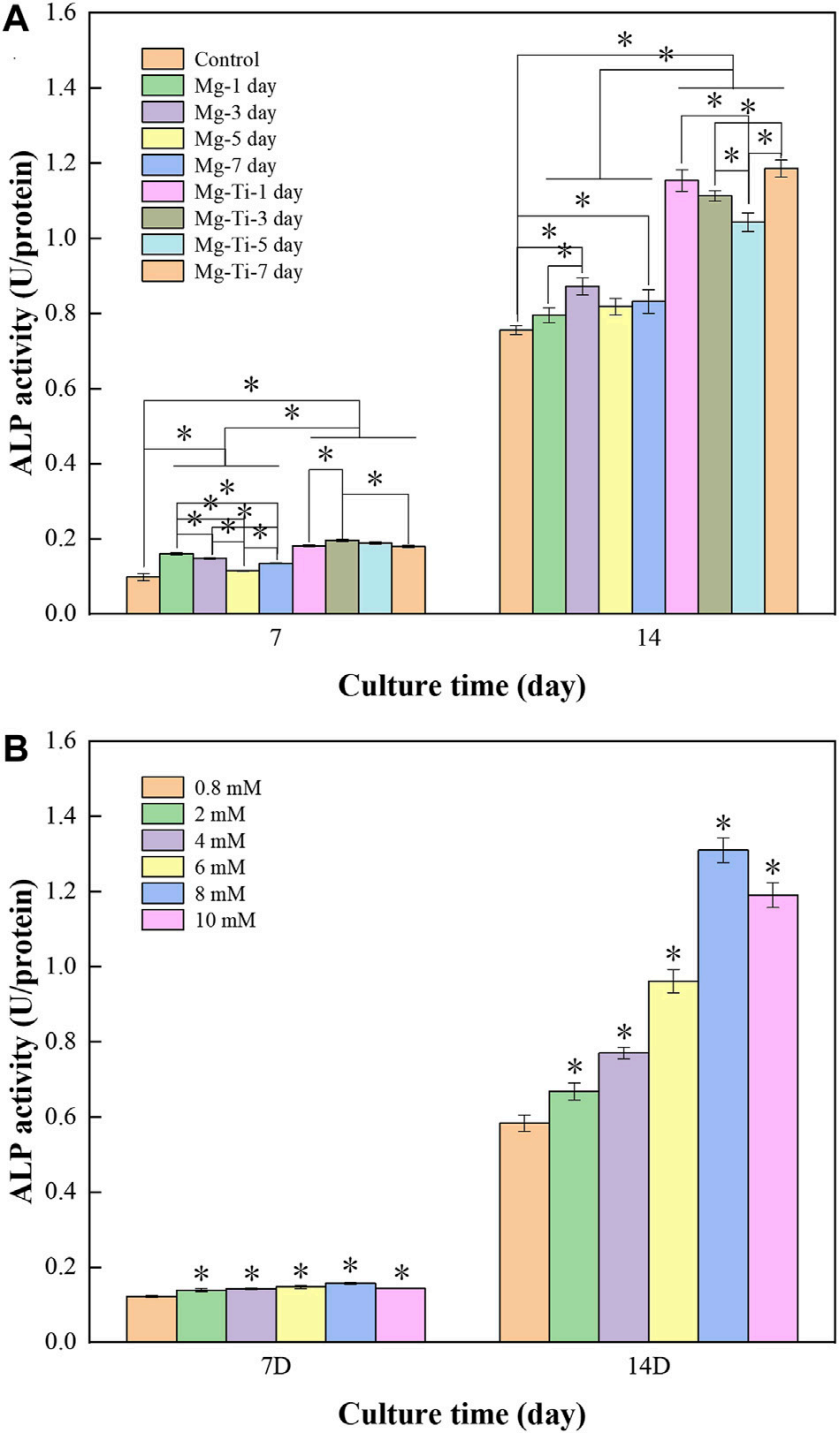
**(A)** ALP activity of MC3T3-E1 cells cultured with extracts of the degraded Mg-Ti composite and pure Mg for 7 and 14 days (n=3 and *p < 0.05). **(B)** ALP activity of MC3T3-E1 cells cultured with gradient Mg^2+^ concentrations for 7 and 14 days (n=3 and *p < 0.05).

The differentiation ability of MC3T3-E1 cells is used to describe the osteoblast maturation and can be assessed by an ALP test. Figure 12A shows that the ALP activity is promoted in the Mg-Ti composite and pure Mg groups on days 7 and 14. It can be found that the ALP content of both groups increased as the osteogenic induction time was extended. On days 7 and 14, the ALP content of the Mg-Ti group is significantly higher than that of the pure Mg group (*p* < 0.05). In the different immersion times of pure Mg and Mg-Ti groups, the results show little difference. Figure 12B shows the ALP activity of MC3T3-E1 cells incubated in a gradient Mg^2+^ concentration medium for 7-days and 14-days. It can be identified that the addition of Mg^2+^ to the medium promotes the ALP activity of cells. The ALP activity is the highest in the 8 mM Mg^2+^ medium within both groups.

## 4 Discussion

### 4.1 Degradation Behavior

As is known, Mg is active and can react with moisture or water when exposed to aqueous environments, resulting in hydroxide ions (OH^−^), hydrogen gas (H_2_), and Mg^2+^ ions by the following reactions (Jin et al., 2020):

$$Mg\;+\;2H_{2}O\;\to \;Mg^{2+}+\;2OH^{-}+H_{2}↑,$$ (1)

$$Mg{\;}^{2+}+\;2OH{\;}^{-}\to \;Mg{\left(OH\right)}_{2}↓.$$ (2)

Generally, the corrosive attack in pure Mg normally starts at the grain boundary. However, galvanic corrosion is induced by the electrode potential differences of Ti and Mg metals in the Mg-Ti composite. The Ti phase is protected as cathodic sites, and the Mg phase is selectively corroded as anodic sites.

Ideal Mg-Ti composites should maintain Ti scaffold integrity to provide the implant strength during the bone defect repair process after the Mg phase degradation. However, as the composites undergo degradation, hydrogen gas accumulation around the cathodic site (Ti skeleton) may cause adverse effects. Different amounts of hydrogen gas could be produced depending on the corrosion rate of the composites. The produced hydrogen could create stresses during corrosion, especially on the Ti skeleton. Moreover, sudden hydrogen gas evolution could also cause the initiation of micro-cracks in the Ti skeleton, resulting in catastrophic failure. Esen et al. (2020) fabricated Ti6Al4V-Mg/WE41/AZ27 composites through powder metallurgy. After immersion for 1-day, the Ti6Al4V-Mg composite samples were not able to preserve their Ti scaffold integrities. The loss of Ti scaffold integrities mainly resulted from the high pressure generated by hydrogen gas. The generated pressure overcame the local strength of sintering necks in the Ti6Al4V alloy skeleton. In the present work, although the degeneration rate of the Mg-Ti composite is higher than that of pure Mg, there is no obvious change in the macro-morphology of the sample with only a few cracks in the Ti regions close to Mg after 7-days of immersion. These all result from the unique design of the 3D interpenetrating phase architecture. By retaining the structural integrity and resisting the development of damage, the mechanical properties of the Mg-Ti composite are enhanced by the interpenetrating phase architecture. The stresses caused by the accumulation of hydrogen gas during the process of corrosion are permitted effective transfer within each bi-continuity of the Ti and Mg phases to generate a substantial strengthening effect in the composite (Zhang et al., 2020).

In this work, the Mg-Ti composite exhibits a higher degradation rate than pure Mg during 7-days of degradation. The high degradation rate might mainly result from galvanic corrosion in the Mg-Ti composite. Because of the electrode potential difference of Ti and Mg metals, Mg with higher activity is preferentially corroded. As shown in Figure 4, the micromorphology image of the Mg-Ti composite shows that the corrosion of Mg regions in the composites preferentially starts from the Mg and Ti interface and gradually extends to the central regions of Mg over time. Apart from galvanic corrosion, the irregularly porous morphology of Mg after preferential corrosion may be another reason for the higher degradation rate. In addition, the irregularly porous corrosion morphology is similar to cancellous bone, thereby stimulating the growth of new bone tissue near the implant.

With regard to the Mg-based materials, the formation of corrosion products is generally characterized by the degradation process. Magnesium hydroxide, generated on the surface of materials, is characterized as the main corrosion product. As shown in Figure 5, the surface of the Mg-Ti composite was almost covered with corrosion product after 7 days of immersion, and the abundance in the Mg site is more than that of the Ti site. The reason for the Mg(OH)_2_ formation promotion might be that Mg^2+^ and OH^−^ accumulate in the Mg region as it is the anodic site in galvanic corrosion. The Mg(OH)_2_ film on the surface of the material could passivate Mg in basic environments as a protective layer and prevents further corrosion underneath Mg. However, the layer could not maintain long-term stability in aqueous environments, especially with the presence of bromide, chlorate, sulfate, and chloride. Soluble MgCl_2_ converted from Mg(OH)_2_ could further transform to magnesium phosphate and finally transform into apatite. This may be the reason why the pH values have remained steady in the immersion test.

### 4.2 *In Vitro* Cytocompatibility of MC3T3-E1 Cells

#### 4.2.1 Cell Viability and Morphology

The reactions between Mg and the biological environment have to be taken into account when Mg-based materials are used as orthopedic implants. In the degradation process, especially at its early stage, the reaction of Mg with water molecules in the aqueous environment leads to the formation of hydroxide ions (OH^−^), hydrogen gas (H_2_), and a high concentration of Mg^2+^. These three factors affect the biocompatibility of Mg-based materials. However, the degradation rate of Mg-based materials *in vivo* is not easy to be simulated by extracts obtained from the current ISO 10993 standards because of the large differences between *in vivo* and *in vitro* conditions. In the study by Gao, Su and Qin (2021), the results suggested that the immersion degradation rate *in vitro* was 2–4 times higher than the degradation rate of the *in vivo* implantation experiment. Similarly, the 1–5 correlation factors of the degradation rate can be predicted between *in vitro* and *in vivo* when an appropriate medium was used for the *in vitro* immersion test (Witte et al., 2006). Sanchez et al. (2015) also reported that the degradation rate *in vitro* reaches up to nine times higher than the *in vivo* degradation. Therefore, Wang et al. (2015)suggested that the extract of Mg-based materials should be diluted 6–9 times to test biocompatibility *in vitro*, according to the selection of implantation.

In this work, the extracts of the samples were diluted six times to evaluate the Mg-Ti composite and pure Mg availabilities for cell morphology, proliferation, and formation of alkaline phosphatase. Then, the Mg^2+^ concentrations of the diluted extracts were measured. These values were used as the basis for preparing culture media with various Mg^2+^ concentrations. As shown in Figure 5, the Mg^2+^ concentration of diluted pure Mg extracts decreases gradually over time, yet that of the Mg-Ti composite remains stable around 8 mM in the process of 7-days of degradation.

In general, the primary requirement of orthopedic implants is to be non-toxic to cells. The CCK-8 method is most commonly used to detect cell proliferation *in vitro*. According to CCK8 results, it can be found that Mg-Ti groups exhibit a similar cell proliferation ability of osteoblasts compared to pure Mg groups in the process of 7-days of degradation. In addition, both pure Mg and Mg-Ti groups have positive effects on the proliferation ability of osteoblasts compared with the control group on days 1, 2, and 3, which indicated that all the extracts in this study are non-toxic and of excellent biocompatibility. Similar results are also obtained after 3 days of culture in media (0.8, 2, 4, 6, 8, and 10 mM Mg^2+^) where the cell proliferation rates of all groups have no significant difference, showing that Mg^2+^ is non-toxic to cells under 10 mM.

In addition, Mg^2+^ also exerts an effect on cellular morphology. An appropriate Mg^2+^ concentration promotes the formation of the actin filament bundle and the expansion of cells, which is beneficial to cell adhesion and motility. It is widely accepted that the F-actin cytoskeletal network is a key regulator of cellular shape and force generation in cell migration with crucial roles in maintaining the cellular shape and elasticity. F-actin staining results (Figure 9) show that cell morphology with more filopodia was observed in the group with an Mg concentration at 8 mM. In the report by Maier and Haraszti (2015), Mg^2+^ has a significant influence on the global structure of the actin filament bundles. The addition of Mg^2+^ contributes to the changes in the elasticity of the actin network even at low concentrations (2–12 mM). Moreover, the speed and extent of bundle formation increase when elevating the Mg^2+^ concentration from 1 to 5–10 mM (Hu and Kuhn 2012). These all suggest that Mg^2+^ around 8 mM is an appropriate concentration benefiting the integration of implants and surrounding tissues. Moreover, as shown in Figure 8, the cell morphology of the Mg-Ti composite is better than that of pure Mg. This may result from the cooperated effect of Mg^2+^ concentration and corrosion products caused by different degradation modes and rates.

#### 4.2.2 Cell Apoptosis

Apoptosis, occurring over several hours, is well known as cell programmed death since death results from the cell itself, eliminating damaged cells (Matsui et al., 2017). The apoptosis rate is an essential index when characterizing the biocompatibility of orthopedic implant materials. In the process of apoptosis, mitochondria play an essential role, which is associated with intracellular Ca^2+^ overload. Larger amounts of Ca^2+^ influx result in intracellular Ca^2+^ concentration rise and accumulation in the mitochondria (Thor, Hartzell and Orrenius 1984). The accumulation of Ca^2+^ contributes to the opening of a high-conductance pore in the inner mitochondrial membrane (IMM), which has been termed mitochondrial permeability transition (MPT) (Douglas et al., 1979). Then, the osmotic swelling of the mitochondria and rupture of the mitochondrial membrane occur which results in the mitochondrial proteins, including cytochrome C, releasing into the cytosol (Lemasters et al., 1999). In the cytosol, an apoptosome complex is formed by cytochrome C together with apoptosis activating factor-1 (Apaf-1) and pro-caspase-9. Cell apoptosis can be triggered by the apoptosome complex through intrinsic or extrinsic apoptosis pathways.

In the experiment of incubating MC3T3-E1 cells with a series of culture media in increasing Mg^2+^ concentrations, the results of the apoptosis analysis implied that the apoptosis rate gradually decreased with the increase in the Mg^2+^ concentration. The minimum apoptosis was observed in the 8-mM Mg^2+^ concentration culture medium. The reasons may be as follows: on one hand, the elevation of extracellular Mg^2+^, as the well-known calcium competing ion, suppresses the increase in the intracellular Ca^2+^ concentration by competing with extracellular Ca^2+^ and inhibiting the release of intracellular calcium, thereby preventing cell damage and reducing apoptosis (Bojan et al., 2013). On the other hand, several *in vitro* studies implied that the elevation of intracellular Mg^2+^ was observed in the early phase of apoptosis (Zhang et al., 2005). However, in the research of Matsui et al. (2017), they developed a novel Mg^2+^ probe which achieved a long-term visualization of intracellular Mg^2+^ dynamics during apoptosis. The results showed that the increase in the Mg^2+^ concentration is associated with the decrease in the ATP concentration after apoptotic cell shrinkage, demonstrating that Mg-ATP is the main resource for the Mg^2+^ increase after cell shrinkage during apoptosis. Moreover, the same conclusion was drawn by Chien et al. (1999) that the elevation of cytosolic free Mg^2+^ was irrelevant to the extracellular Mg^2+^ concentration with the increase in intracellular Mg^2+^ derived from the mitochondria during the process of apoptosis. Together, Mg^2+^ concentration at an appropriate range tends to decrease the apoptosis rate of cells. A recent study has documented that the addition of Mg particles reduces the apoptosis caused by the Ti particles (Wang Y et al., 2020). However, the apoptosis results of experimental extracts were not completely consistent with the aforementioned trends. It might be attributed to the generation of other factors (pH, osmolality, and corrosion product) in the degradation process.

#### 4.2.3 Cell Differentiation

ALP, as the key factor governing the process of osteogenesis, is expressed in the early stage of bone development and used as an early marker of osteoblast differentiation (Hu et al., 2021; Yi et al., 2021). In the present study, the ALP activities of the Mg-Ti and pure Mg groups with different degradation times were both higher than those of the control group. In addition, the ALP activity gradually decreased over time, which was consistent with the Mg^2+^ concentration trend of pure Mg extracts. It showed that the extracts of Mg-based materials could enhance the ALP activity of cells and the enhancing efficiency was closely related to the Mg^2+^ concentration of extracts. Moreover, the ALP activity of Mg-Ti groups was higher than that of pure Mg groups at all degradation times, which indicated that the extracts of the Mg-Ti composite could better promote the osteogenic differentiation of cells compared with pure Mg. The difference in the ALP activity of the two materials might be attributed to the following aspects. On one hand, the Mg^2+^ concentration of Mg-Ti composite extracts remained at around 8 mM in 7-days of degradation, which is regarded as an appropriate range for cell osteogenic differentiation. This assumption was verified by the experimental result of MC3T3-E1 cells incubated with varying Mg^2+^ concentrations. As shown in Figure 12, the ALP activity of the 8-mM group was significantly higher than that of the other five groups with the difference being statistically significant. On the other hand, the Mg-Ti composite exhibited a slightly higher pH value of less than 8.5 than that of pure Mg in the process of degradation. It was reported that suitable alkalinity (pH 7.8–8.5) had a beneficial effect on osteogenesis (Liu et al., 2016; Wu et al., 2019). However, the 7-day extract groups showed a similar Mg^2+^ concentration to 5-days, whereas they exhibited a higher ALP activity. This phenomenon may result from the production of more calcium phosphate after 7-days of degradation, which improves the osteogenic ability of cells.

In summary, the extracts of the Mg-Ti composite exhibit good biocompatibility and excellent osteogenic activity in the process of 7-days of degradation. However, because of the difference in degradation rates *in vitro* and *in vivo*, animal studies need to be carried out in the future to determine the suitability of the Mg-Ti composite as an orthopedic implant.

## 5 Conclusion

1) A new Mg-Ti interpenetrating phase composite is fabricated by printing a pure Ti scaffold through the 3D printing technology and then the pressureless infiltration of the Mg melt into it. The degradation of the Mg-Ti composite starts from the Mg matrix near the interface of the Ti and Mg region. The Ti-based skeleton remained intact, while Mg processively degraded during different periods. In the degradation process of the Mg-Ti composite, a slightly alkaline environment is formed which is conducive to the cell culture.

2) The extracts of the Mg-Ti composite were showed to be non-toxic to cells during 7 days of degradation. Compared with pure Mg extracts, it exhibited better cell morphology and higher osteogenic activity. Mg^2+^, which was precipitated during degradation, had an impact on cell apoptosis, and the rates of apoptosis were overall within an acceptable range.

## Data Availability

The original contributions presented in the study are included in the article/Supplementary Material; further inquiries can be directed to the corresponding author.
